# Elongated magnetite nanoparticle formation from a solid ferrous precursor in a magnetotactic bacterium

**DOI:** 10.1098/rsif.2016.0665

**Published:** 2016-11

**Authors:** Jens Baumgartner, Nicolas Menguy, Teresa Perez Gonzalez, Guillaume Morin, Marc Widdrat, Damien Faivre

**Affiliations:** 1 Department of Biomaterials, Max Planck Institute of Colloids and Interfaces, 14424 Potsdam, Germany; 2 Institut de Minéralogie et de Physique des Milieux Condensés, Unité Mixte de Recherche 7590 Centre National de la Recherche Scientifique, Université Pierre et Marie Curie, Institut de Recherches pour le Développement, Campus Jussieu, 75005 Paris, France

**Keywords:** magnetite, magnetotactic bacteria, ferrous iron, biomineralization, precursor

## Abstract

Magnetotactic bacteria are aquatic microorganisms that intracellularly mineralize ferrimagnetic nanoparticles enabling the cells to align with the geomagnetic field. The bacteria produce a magnetic mineral of species-specific phase (magnetite Fe(II)Fe(III)_2_O_4_ or greigite Fe(II)Fe(III)_2_S_4_), size, morphology and particle assembly. Several species produce crystals of unusual elongated particle shapes, which break the symmetry of the thermodynamically favoured isometric morphology. Such morphologies are thought to affect domain size and orientation of the internal magnetization. Therefore, they are interesting study objects to develop new synthetic strategies for the morphological control of nanoparticles. We investigate the formation of such irregularly shaped nanomagnets in the species *Desulfovibrio magneticus* RS-1. In contrast to previously described organisms, this bacterium accumulates iron predominantly as Fe(II) rather than Fe(III) consistent with an alternative oxidative biomineralization route. Further, using high-resolution electron microscopy, we observe an epitaxial relationship between precursor and the final mineral phase supporting the notion of a solid-state transformation pathway. The precursor is likely a green rust previously thought to convert to magnetite only by dissolution and re-precipitation. Our findings represent a novel observation in the interconversion of iron (oxyhydr)oxide materials and suggest that solid-state growth processes could be required to produce irregularly shaped, elongated magnetite nanocrystals.

## 1. Background

The ferrimagnetic iron oxide magnetite (Fe_3_O_4_) is known to be biomineralized by the so-called magnetotactic bacteria, a diverse group of aquatic microorganisms with the ability to navigate by magnetotaxis, i.e. a movement directed by the geomagnetic field [1,2]. This ability is enabled by the formation of the mineral as nanometric, stable single magnetic domain particles of species-dependent size and morphology within intracellular compartments (magnetosomes). To sum the individual particles' magnetic dipoles, these structures are usually aligned in a chain-like fashion along the longitudinal cell axis, providing directionality in a chemotactic swimming behaviour in search for optimal oxygen and nutrient conditions within a stratified water column [3].

Magnetite biomineralization has also been observed in birds and fish, where it is suspected to play a similar role in geomagnetic field sensing and in chitons (*Polyplacophora,* maritime molluscs), where it serves a mechanic function by hardening of the animal's teeth that are used to scrape algae from marine rocks when feeding [4–7]. In aqueous solution under ambient conditions, magnetite forms either by co-precipitation of ferrous and ferric iron in a stoichiometric ratio of 1 : 2, by reductive phase transformation of a ferric iron oxyhydroxide precursor such as ferrihydrite or by oxidation of a precursor high in ferrous iron content such as green rust [8–10]. Owing to the aqueous solvation, biological formation processes may be expected to follow similar schemes.

Consistently, previous observations in magnetotactic α-*Proteobacteria* grown under (micro)oxic conditions have shown that magnetite forms from nanometric, poorly ordered ferric iron oxyhydroxides such as ferrihydrite, while oxidative routes have not yet been observed [11–15]. However, the investigated species produce only isometric or prismatic particles, while surprisingly some magnetotactic bacterial strains are able to break the cubic crystal symmetry of magnetite and form irregularly shaped particles with a different preferential magnetization axis (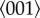 instead of 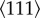) [16–21]. Through which process such thermodynamically unfavourable habits can arise remains unclear. In addition to templating by the organic components surrounding the particles (lipid membrane and proteins) [22], crystallization from metastable amorphous or crystalline solids of a different precursor as well as oriented attachment of previously formed subunits could provide a path to such particles [23]. However, neither solid-state transformations nor oriented attachments have been observed in bacteria with unusual particle morphologies.

To address the question how magnetotactic bacteria are able to produce anisotropic magnetite particles, we investigated the biomineralization of magnetite in the δ-proteobacterium *Desulfovibrio magneticus* RS-1. This strain has been studied earlier by Byrne *et al.* with a focus on intracellular compartimentalization and on the formation of amorphous Fe-P granules that are presumably distinct and unrelated to magnetite mineralization in the bacteria [24]. Here, we focus on the iron speciation in the bacteria grown under anaerobic conditions and the mineralization precursors to magnetite. We show that the crystallization of magnetite in this organism involves precursors that differ chemically and structurally from previous observations in isometric or prismatic particle producers. We find that a solid ferrous iron phase serves as precursor to magnetite and that this transformation likely occurs via a solid-state transformation, a so-far overlooked link between the two minerals.

## 2. Material and methods

### 2.1. Bacterial samples

*Desulfovibrio magneticus* (RS-1) was obtained from Deutsche Sammlung von Mikroorganismen und Zellkulturen GmbH (DSMZ 13731; JCM 12223) [25]. For induced magnetite mineralization experiments, *D. magneticus* was grown anaerobically with pyruvate as electron donor and fumarate as electron acceptor at 28°C in a medium composed of 6 mM potassium monophosphate, 5 mM ammonium chloride, 10 mM fumaric acid disodium, 10 mM sodium pyruvate, 1.2% (v/v) Wolfe's minerals without sulfate and iron, and 285 µM cysteine-HCl until cells were devoid of particulate iron. Sulfate is omitted to avoid respiration to sulfide that hampers the uptake of iron and magnetite production [24]. Also note that when fumarate is depleted, pyruvate may also be fermented [25]. Iron is present in the medium predominantly in the reduced, ferrous form as determined by ferrozine assay (Fe(II)/Fe(III) = 2) [26]. To induce magnetite biomineralization, cells at OD_575 nm_ = 0.2 (after 4 days of growth) were transferred to iron-spiked medium (i.e. with 100 µM ferric citrate added). Control cultures were grown continuously in the presence of iron. For X-ray absorption spectroscopy (XAS), cell cultures were sampled (after *t* = 0, 3, 25, 56, 74 and 120 h with OD_575 nm_ = 0.2, 0.3, 0.4, 0.9, 0.9, 0.9, respectively) in batches of 90–135 ml each, centrifuged 5 min at 8000 r.p.m., 4°C and washed three times with 5 ml Tris-buffered saline (TBS, pH 7.6). The pellets were re-suspended in 100 µl TBS plus 25 µl glycerol and frozen in liquid nitrogen on sample holders with Kapton film support. Samples were stored at −80°C, sent to ESRF on dry ice, where they were stored at −80°C until measurement.

### 2.2. Electron microscopy

Cells were adsorbed from suspension to carbon film-coated Cu mesh grids and rinsed with deionized water. Low-resolution transmission electron microscopy was performed on a Zeiss EM 912 Omega with 120 kV acceleration voltage. High-resolution transmission microscopy (HRTEM), high angle annular dark field-scanning transmission microscopy (HAADF-STEM), and energy dispersive X-ray spectroscopy (XEDS) elemental mapping were carried out on a Jeol 2100F microscope. This machine, operating at 200 kV, is equipped with a Schottky emission gun, an ultra-high-resolution pole piece, and an ultrathin window JEOL detector. HRTEM images were obtained with a Gatan US 4000 CCD camera.

### 2.3. High-resolution transmission microscopy analysis

Reflection intensities were mapped by generating fast Fourier transforms (FFTs) of approximately 2.5 × 2.5 nm image areas using ImageJ and recording intensity counts at the specific reflection position as a function of each partial image. Heatmaps show normalized reflection intensity differences over the whole image. Variations in the reflection intensity within a particle indicate discrete regions with varying crystallinity and orientation. Stereographic projection analysis was performed using SingleCrystal 2 (http://www.crystalmaker.com/singlecrystal/index.html) and crystal morphology modelling was achieved using KrystalShaper (http://www.jcrystal.com/products/krystalshaper/).

### 2.4. X-ray absorption spectroscopy

The Fe K-edge X-ray absorption near edge structure (XANES) was recorded at beamline ID26 (ESRF). Samples were cooled to around 20 K using a liquid He cryostat. We used an Si(111) double-crystal monochromator and focusing mirrors to obtain a beam spot size of approximately 200 × 400 µm^2^ on the samples. Data were recorded in fluorescence detection mode using a spectrometer with five spherically bent analyser crystals. XANES spectra were recorded with approximately 0.1 eV resolution from 7100 to 7200 eV. Recorded spectra were averaged using PyMca 4.7.4 after evaluation for photo-reduction and other artefacts [27]. Spectral normalization and XANES data linear combination fitting were performed with Demeter 0.9.20 [28]. The pre-edge was analysed after background subtraction of an error function fitted to the edge step, with the centroid being determined as 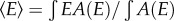 and the area 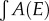, where *E* is the energy and *A* the normalized absorbance.

### 2.5. Reference compounds for X-ray absorption spectroscopy

Nanoparticulate hematite (Hm) was obtained from Alfa-Aesar, goethite (Gt) and spinach ferredoxin (Fe-S) from Sigma-Aldrich. All other samples were prepared on site and measured as frozen suspensions. Magnetite (Mt), ferrihydrite (Fh) and the prokaryotic ferritin proxy ferric phosphate hydroxide (Fe(III)-P 2/3) were prepared as described earlier [11]. Mt was precipitated under nitrogen atmosphere by slow dosing of a ferrous and ferric iron chloride solution (333 mM Fe(II) and 667 mM Fe(III) at a rate of 1 µl min^−1^) to a dilute sodium hydroxide solution (10 ml) constantly kept at pH 9. Fh was precipitated by neutralization of a ferric nitrate solution (0.1 M) with sodium hydroxide. Fe(III)-P 2/3 and ferrous phosphate (Fe(II)-P 3/2) were precipitated analogously under nitrogen after the addition of phosphoric acid with stoichiometries of Fe/P = 2/3 and 3/2, respectively. Ferrous polyphosphates (Fe(II) triP and Fe(II) hexaP) were synthesized by adding 1 ml of a 200 mM ferrous chloride solution to either 1 ml of 33 mM sodium hexametaphosphate or 40 mM sodium triphosphate pentabasic and neutralization with NaOH under nitrogen atmosphere. All solutions were prepared from nitrogen sparged deionized water to avoid oxidation. Ferric polyphosphates (Fe(III) triP and Fe(III) hexaP) were synthesized by addition of 1 ml of a 200 mM ferric chloride solution to 1 ml of either 50 mM sodium hexametaphosphate or 60 mM sodium triphosphate pentabasic and neutralization with NaOH. Green rust II (GR) was synthesized under argon by neutralizing a solution of 150 mM (NH_4_)_2_Fe(SO_4_)_2_·6H_2_O and 50 mM Fe_2_(SO_4_)_3_ with sodium hydroxide. Phosphate-intercalated green rust was prepared according to a procedure adapted from Hansen and Poulsen by mixing of the GR with a 200 mM disodium phosphate solution under nitrogen for 1 day [29].

## 3. Results

Nascent magnetosome particles are only rarely found in conventionally grown cultures or environmental isolates. Therefore, to increase the probability to observe particles during the formation process, we employed a previously developed method of iron starvation followed by transfer to iron-spiked cell culture medium [12,24]. We performed HRTEM investigations and XEDS elemental mapping on cells during the process to localize and characterize the intracellular iron mineral phases. The magnetic response of the cells was determined by the magnetically induced differential light-scattering coefficient (*C*_mag_) assay to assess the stage of magnetite formation in the bacterial culture [30].

**Figure 1.**
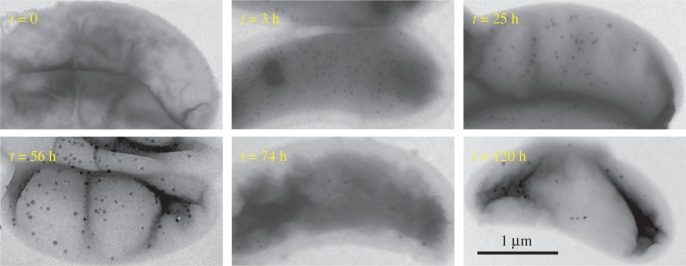
Bright field transmission electron micrographs of RS-1 cells at *t* = 0, 3, 25, 56, 74, 120 h after transfer to the iron spiked medium. (Online version in colour.)

**Figure 2.**
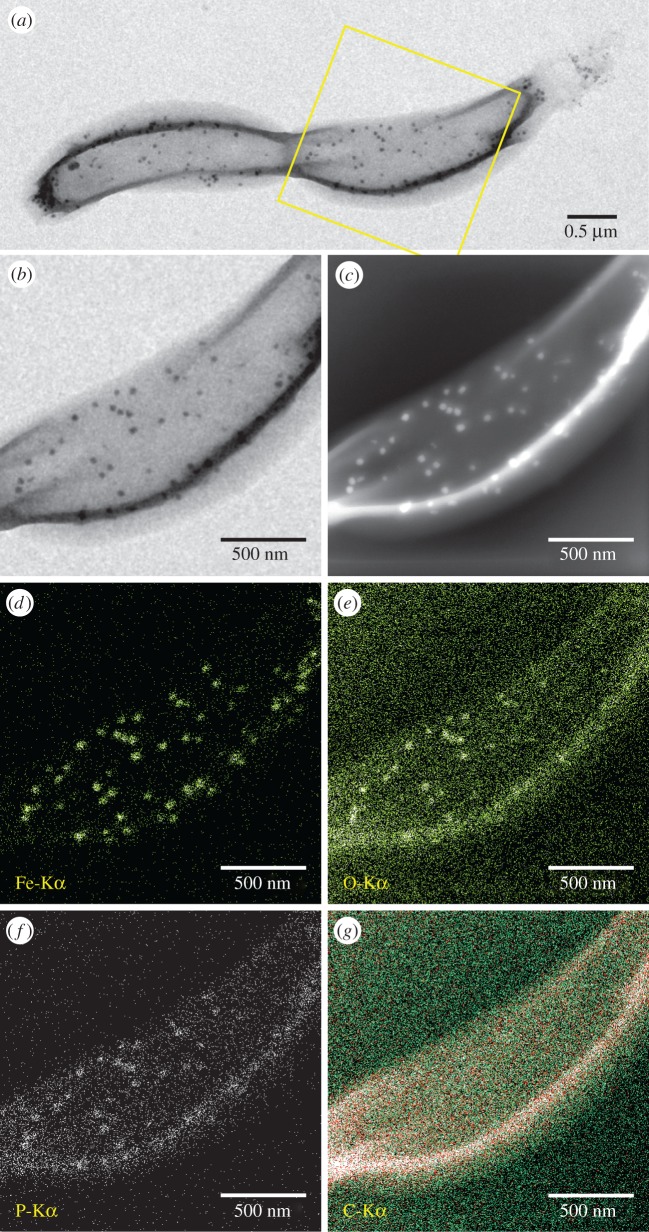
Elemental analysis of *D. magneticus* (RS-1) cells with Fe-P granules (*t* = 25 h). (*a*) Bright field TEM image with marked region in (*b*), (*c*) corresponding STEM-HAADF image, (*d–g*) XEDS elemental maps of Fe, O, P and C. Sensitivity of XEDS is of the order of 1 at%. (Online version in colour.)

**Figure 3.**
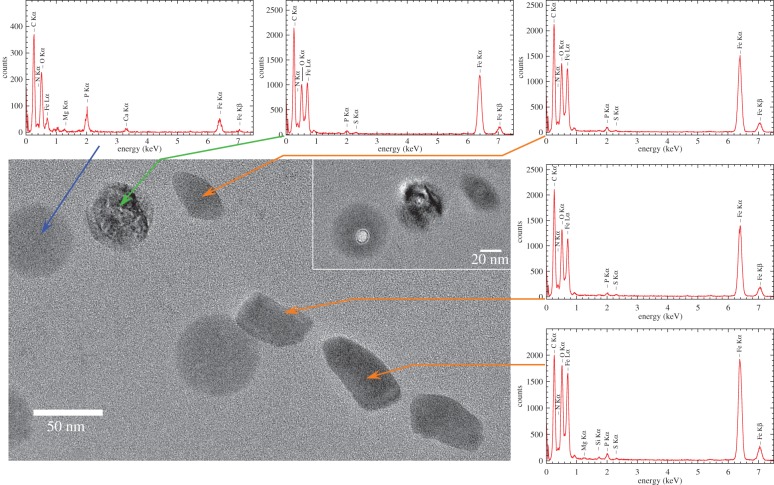
TEM image of region containing Fe-P granules and a chain of elongated mature magnetite particles and associated elemental composition (*t* = 56 h). XEDS spectra of magnetite particles (orange arrows), Fe-P granule (blue arrow) and potential intermediate (green arrow). Inset: beam damage after XEDS measurements indicates damage to Fe-P granule and presumed intermediate, but not magnetite.

We first ensured that iron-starved cells were devoid of any iron-containing solid inclusions (figure 1, *t* = 0) and did not exhibit any kind of magnetic response (*C*_mag_ = 0). Upon addition of iron to the medium, the bacteria accumulated iron and intracellularly formed amorphous granules with an elemental composition dominated by Fe, P and O (figure 1, figures 2 and 3) as described earlier by Byrne *et al.* [24].

**Figure 4.**
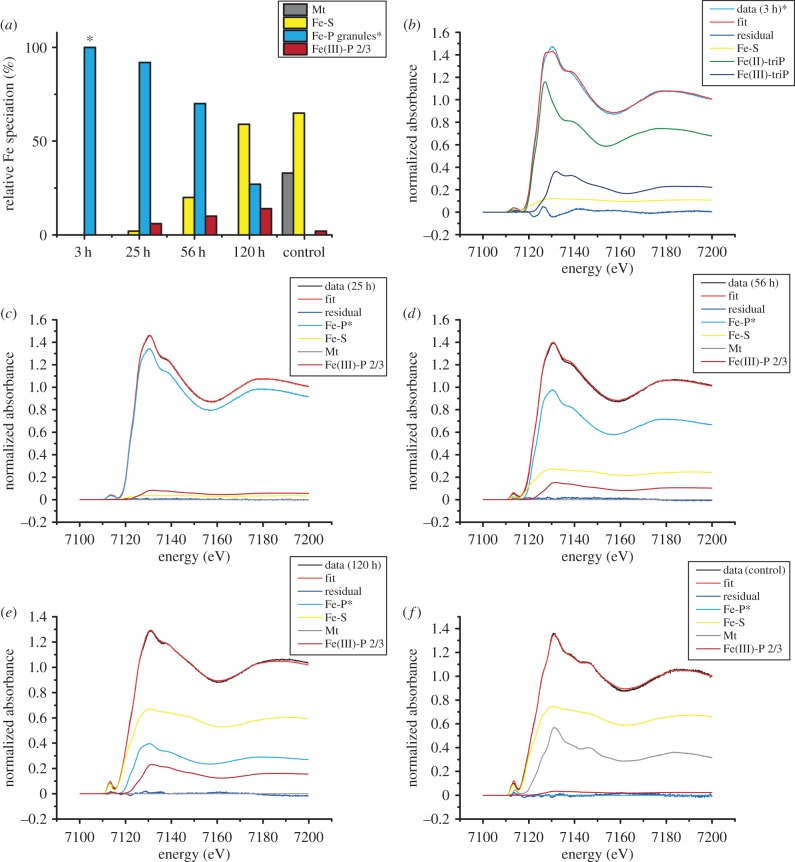
Bulk Fe speciation determined by Fe K-edge XAS on *D. magneticus* RS-1 in different stages. (*a*) Relative contributions of different iron pools over time and in control cell culture. Magnetite (Mt), Fe-S (spinach ferredoxin), amorphous Fe-P granules and ferritin (Fe(III)-P 2/3). (*) Note that combinatorial fitting yielded only unsatisfactory fits for cells in the earliest stage that are dominated by Fe-P granules (see large residual in (*b*)). Therefore, in further stages the spectrum of the Fe-P granules is approximated by the cellular spectrum after 3 h. (*b*) *t* = 3 h, (*c*) *t* = 25 h, (*d*) *t* = 56 h, (*e*) *t* = 120 h, (*f*) control culture; Mt shows a characteristic shoulder feature in the adsorption edge at 7127 eV and strong peaks at 7131 and 7146 eV.

**Figure 5.**
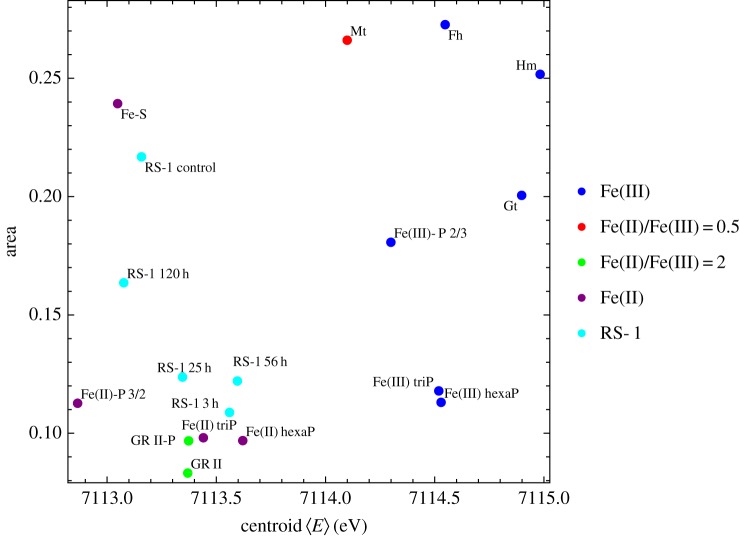
Fe K pre-edge peak area versus centroid position. The mean centroid position of all bacterial samples (RS1) is shifted with respect to the mean Fe(III) compounds by −1.3 eV and cluster with pure Fe(II) and Fe(II)/Fe(III) = 2 compounds. This indicates a predominantly ferrous iron state in the investigated bacterial samples. Mt, magnetite; Fh, ferrihydrite; Hm, hematite; Gt, goethite; Fe-S, spinach ferredoxin; GR II, green rust; GR II-P, phosphate intercalated green rust; Fe(II)/Fe(III) triP, iron triphosphates; Fe(II)/Fe(III) hexaP, iron hexaphosphates; Fe(II)-P 3/2, ferrous phosphate with Fe/P ratio of 3/2; Fe(III)-P 2/3, ferric phosphate hydroxide with Fe/P ratio of 2/3—a proxy of bacterioferritin.

To determine the phase and oxidation state of Fe in the granules, we performed Fe-K edge XAS on frozen cell pellets after iron spiking of the medium. After 3 h, the cells had formed many Fe-P granules that were dispersed throughout the cells (figure 1, *t* = 3 h). Yet, according to TEM and *C*_mag_, they were still devoid of magnetite and therefore no contribution of magnetite is expected in the XAS spectra. We compared the spectral near edge structure (XANES) by combinatorial linear combination fitting to numerous biologically relevant reference compounds including different iron (oxyhydr)oxides, Fe(II) and Fe(III) phosphates and polyphosphates, green rusts (pure sulfate and phosphate intercalated) and the Fe-S cluster species in ferredoxin (from spinach sp.). Unfortunately, we were not able to obtain fits of sufficient quality that describe the XANES spectra of the Fe-P granules satisfactorily. Best fits were obtained by a combination of Fe(II) triphosphate (68%), Fe(III) triphosphate (21%) and Fe-S (11%); however, large residuals show that the structure significantly deviates from the model compound combination (figure 4). Hence, to gain more insight into the redox chemistry, we analysed the centroid position of the pre-edge peak feature that correlates with the oxidation state of iron and its local coordination environment [31]. The centroid is shifted by −1.1 eV with respect to the average positions of Fe(III) reference compounds, by −0.5 eV with respect to magnetite (Fe(III) : Fe(II) = 2), close to green rust (+0.2 eV, Fe(III) : Fe(II) ≈ 0.5) and Fe(II) polyphosphates (+0.03 eV) indicating an excess of ferrous over ferric iron (electronic supplementary material, figure S1; figure 5). Therefore, under the electron microscopy and elemental mapping supported assumption (figure 2) that the XANES spectrum is largely dominated by the contribution of amorphous Fe-P granules, we deduce that the granules are composed of an iron phase similar either to ferrous polyphosphates or to mixed valence iron polyphosphates with excess of ferrous over ferric iron.

The onset of cellular magnetic response (*C*_mag_ = 0.01) occurred 25 h after cell transfer to Fe spiked medium. At this stage, the cells had formed the first magnetite crystals in addition to the still prevalent Fe-P granules. The bacterial culture's magnetic response reached a plateau at *C*_mag_ = 0.11 within 56 h. In our experiments, the number of Fe-P granules reduced over time (figure 1) as has been demonstrated earlier [24]. However, cells never reached complete depletion and retained some granules over the investigated time range up to 120 h (figure 1, *t* = 120 h). Cells that were grown in regular medium and continuously exposed to Fe occasionally also showed structures in TEM that resemble Fe-P granules (electronic supplementary material, figure S2). The XANES spectrum of these control cells (figure 4*b*) can be described by linear combination of iron in some biomolecular Fe-S form (65%) and magnetite (33%) with a possible contribution of ferric phosphate hydroxide (Fe(III)-P 2/3, 2%) that serves as a proxy for prokaryotic ferritin [11]. The XANES spectra of induced cells from 25 h to 120 h are consistent with a gradual accumulation of Fe-S and ferritin over time at the expense of Fe-P. On the basis of TEM, magnetite appears to represent only a minor fraction of the total intracellular Fe also in the later stages of induction. Therefore, it could not be reliably detected by XANES, for which the detection limit of a minor phase in a mixture is approximately 5% [32].

**Figure 6.**
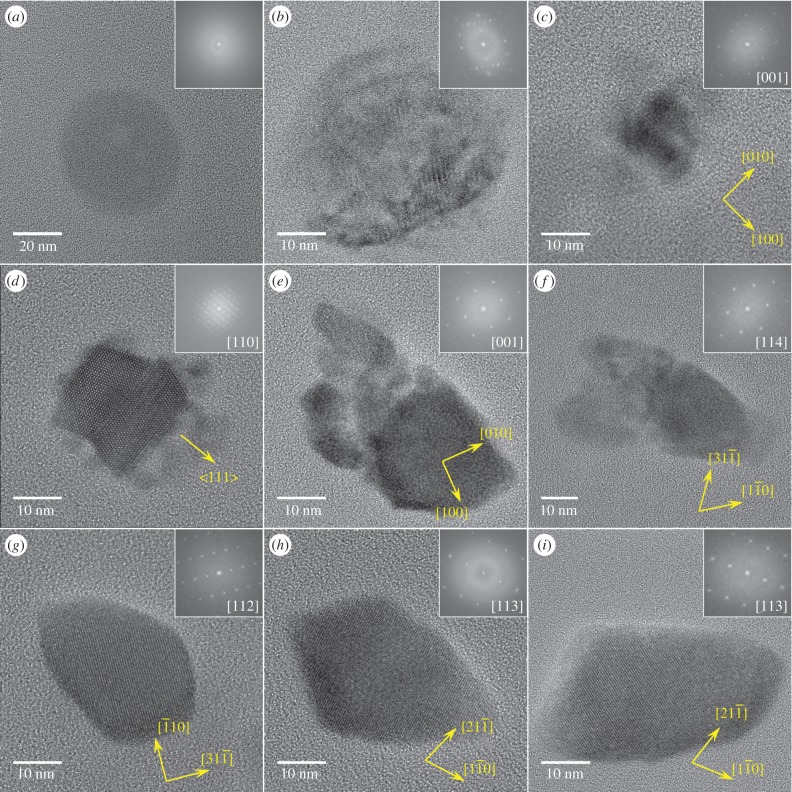
Particulate iron phases in *D. magneticus* (RS-1). Insets: FFTs with indicated zone axis (normal to image plane). (*a*) Amorphous Fe-P granule (*t* = 120 h); (*b*) polycrystalline particle with spherical morphology similar to Fe-P granule, but partial magnetite crystal structure (*t* = 56 h), see the electronic supplementary material, figure S3, for further analysis; (*c*) small magnetite particle with surface-bound secondary phases (*t* = 56 h); (*d*) magnetite twin crystal with surface-bound secondary phases, see figure 7 and the electronic supplementary material, figure S4, for further analysis; (*e,f*) aggregate-like magnetite particles (*t* = 56 h) with similar to mature (*g–i*) elongated morphology (*t* = 120 h). (Online version in colour.)

Over the time course of induced mineralization, we observed several particles by HRTEM with incomplete morphologies (figure 6*b–f*) suggesting different stages in the formation to mature bullet-shaped, single crystalline magnetosomes (figure 6*g–i*). For example, the particle shown in figure 6*b* is polycrystalline, composed of magnetite and at least one other phase that shares a common crystallographic direction along the [110] direction of magnetite (electronic supplementary material, figure S3). The particle was found in proximity to Fe-P granules and mature magnetosomes (figure 3). The chemical composition resembles that of the magnetosomes (figure 3; high Fe, low P concentration), while the quasi-spherical morphology and size resembles amorphous Fe-P granules (figure 6*a,b*) suggesting that granules can transform to magnetite.

**Figure 7.**
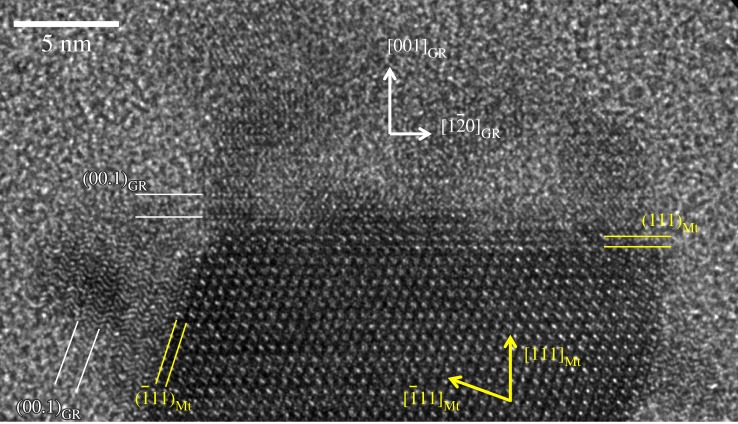
Surface of magnetosomal magnetite (Mt) crystal with epitaxial phase consistent with green rust (GR). (00.1) planes of the GR phase are parallel to the (111) planes of the Mt crystal, zone axis of GR (210) is parallel to Mt (110) (both normal to image plane). (Online version in colour.)

Some small magnetite particles had poorly ordered phases/particles in proximity or attached to their surface that could act as solid growth precursors for the magnetosome particles (figure 6*c,d*). Of particular interest is a twinned magnetite crystal onto which another phase is attached in evident surface contact (figures 6*d* and 7).

The mineralogical identification of this phase is not straightforward. To determine potential phase candidates, we compared simulated small area electron diffraction patterns of various iron (hydr)oxides and (oxyhydr)oxide phases to the FFTs of the observed micrographic data (electronic supplementary material, figures S4 and S5). While the patterns cannot be unique, the interfacial contact between magnetite and the unknown phase, as well as the angular dependencies between both minerals' lattices indicate an epitaxial relationship between them. Here, epitaxy refers to mutually shared crystal orientations as defined by the two-dimensional lattice at their interface where the atomic positions have to be coherent for both phases. The required coherence in the lattice reduces the list of candidates for which such an overlap is possible. We found two phases for which we could determine such an overlap in the lattice: GR II and ɛ-Fe_2_O_3_ (figure 7; electronic supplementary material, figure S5).

ɛ-Fe_2_O_3_ is a rare ferric iron oxide phase that is reportedly formed under unusually harsh chemical conditions, for example, by pyrolysis inside heat-treated samples of infiltrated mesoporous silica [33–35]. Therefore, it is a less likely candidate although the same crystallographic relationship with magnetite has been demonstrated in synthetically produced nanowires by pulsed laser deposition [36]. By contrast, green rusts are known to be produced by reducing [37] or oxidizing [38] microbial activities and have been shown to convert to magnetite by oxidation, presumably through dissolution and re-precipitation [39]. Furthermore, the Fe(II)/Fe(III) bacterial redox chemistry of *D. magneticus* RS-1 observed by XANES analysis appears consistent with the possible presence of minute amounts of green rusts. The phase is a mixed valence ferrous and ferric iron hydroxide with brucite-type planar Fe(OH)_6_ sheets intercalated by a hydrated layer of varying anion composition. The interplanar spacings observed by HRTEM (figure 3) are consistent with the trigonal lattice of GR II (space group 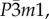 No. 162) with unit cell parameters of *c* ≈ 9.7 Å and *a* ≈ 5.1 Å observed from the (210) zone axis. Common GR II is intercalated by a hydrated sulfate anion layer and has a *c*-axis length of 10.9 Å and *a*-axis length of 5.5 Å [29]. However, the variability in anion composition and hydration levels in green rusts has a large impact on the interlayer distance that can vary between 7.4 Å and 11.2 Å [29,40]. The hexagonal brucite-type lattice (along *a*-axis) is also expected to shrink or expand as a function of the oxidation state and hydroxylation [41] in order to match the underlying substrate lattice (electronic supplementary material, figure S6). In the TEM micrographs (figure 7), the layers in the GR II stack along the *c*-axis parallel to the 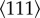 directions of magnetite that correspond to the hexagonal oxygen anion layers stacked in ABCABC sequence enabling the lattice fit.

Furthermore, we observed larger magnetite particles with similar size, but irregular shape resembling mature bullet-shaped morphologies that appear to be composed of multiple grains with either identical or closely related crystallographic orientation (figure 6*e*,*f*; electronic supplementary material, figure S7). Such aggregate structures are often explained in the literature by concepts such as oriented attachment or mesocrystal formation [42–44], i.e. independently formed crystal units which subsequently orient relative to each other and fuse. However, given our observation of secondary phases, such structures could possibly also derive from a gradual transformation of the precursor.

The regular mature products are magnetite particles exhibiting bullet-shaped elongated morphologies primarily along a crystallographic [001] direction (figure 6*g–i*; electronic supplementary material, figure S8). Note that these magnetosomes exhibit similar features as other bullet-shaped crystals, i.e. a large {111} or a double triangle shape associated with a more or less pronounced 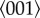 elongation as previously observed for *Nitrospirae* or δ-*Proteobacteria* [18,19].

Finally, at the mature stage, the elongated magnetite particles tend to form short chains preferentially oriented along the elongated particle axis (electronic supplementary material, figure S9) consistent with an earlier observation by X-ray diffraction texture analysis [45].

## 4. Discussion

**Figure 8.**
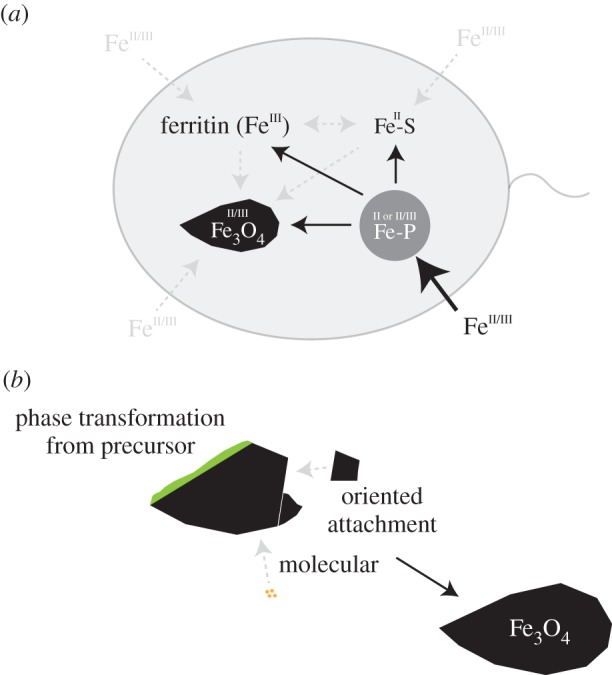
Schematic overview of the observed processes. (*a*) Cellular iron pools and potential connecting pathways. Cells rapidly accumulate ferrous iron in amorphous Fe-P granules (bold arrow) that can likely convert to magnetite and other iron pools, mostly Fe-S (solid arrows); (*b*) magnetite in RS-1 grows from precursors and potentially other pathways involving molecular or oriented attachment mechanisms. (Online version in colour.)

We show that biomineralization of magnetite in *D. magneticus* RS-1 is preceded by the precipitation of a solid ferrous precursor that differs from previous observations in magnetotactic α-*Proteobacteria* and other organisms such as the chiton that produce isometric particles (figure 8*b*). This precursor and the intracellular Fe redox chemistry indicate an oxidative magnetite formation pathway. These findings corroborate those on the abundance of redox-regulating proteins in magnetotactic bacteria [46]. The formation of elongated magnetite particles appears to result from a solid-state transformation of the precursor with growth along the oxygen stacking direction (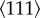) in the magnetite crystal.

Furthermore, our results provide a link between the different intracellular iron pools observed in RS-1: *D. magneticus* RS-1 has four major intracellular iron pools (amorphous Fe-P granules, magnetite, Fe-S and ferritin; figure 8*a*). Iron is most rapidly accumulated in the Fe-P phase that is either purely or largely in the ferrous state (bold arrow, figure 8*a*). Over time, iron is increasingly present in the other iron pools with a strong preference for ferrous biomolecular Fe-S. Electron microscopy indicates that Fe-P granules can convert to magnetite; however, the wide abundance of the granules at the early stages, but low abundance at late stages in correlation with the observed increasing iron contributions in the Fe-S and ferritin pools suggest that most iron is transferred to those (solid arrows, figure 8*a*). It has been claimed earlier that iron from the amorphous Fe-P granules either does not contribute to magnetite formation or does so only in minor amounts [24]. Our findings are consistent with the latter scenario as iron appears to be predominantly contributing to the other iron pools. It is unclear whether further pathways from iron uptake to the different pools as well as other conversions exist (dashed arrows, figure 8*a*).

Note that non-magnetotactic *Desulfovibrio* spp. can produce intracellular polyphosphate granules devoid of iron [47]. This raises the question whether common biochemical pathways to Fe-P granule production in RS-1 lead to their formation. However, beyond the chemistry, the particles observed in RS-1 are significantly different in size and intracellular distribution from other species. While the Fe-P granules in RS-1 are in the size range of magnetosomes and abundantly dispersed throughout the cell, polyphosphates in, for example, *D. gigas* are large granules (hundreds of nanometres in diameter) and singular [47]. In addition, the function of Fe-S formed inside RS-1 remains unclear. Given the large amount of resorbed iron, one could argue that complexation of iron in Fe-S proteins serves as a storage or detoxification mechanism for the cells. Future comparative experiments on different *Desulfovibrio* spp. may resolve the role of both structures.

## 5. Conclusion

The observation of a ferrous precursor to magnetite in *D. magneticus* RS-1 indicates a potential link of the environmental redox conditions of the bacterial habitat and the bacteria's physiology to the applied pathway. Reductive routes could be favoured for bacteria (e.g. magnetospirilla) living under microoxic conditions (where iron is mostly ferric), whereas oxidative routes could dominate for species such as RS-1, living in anoxic conditions (where iron is mostly ferrous). To determine whether the specific cellular iron chemistry of *D. magneticus* RS-1 and/or the solid-state transformation are linked exclusively to the morphology of the particles or only represent a feature of the cellular Fe physiology, will require the study of further strains that produce anisotropic magnetite particles. We envision this knowledge to enable new strategies for the synthetic production of such magnetic nanoparticles.

## Supplementary Material

Electronic Supporting Material
