# Supplementary material for: Elongated magnetite nanoparticle formation from a solid ferrous precursor in a magnetotactic bacterium
Source: J R Soc Interface. 2016 Nov;13(124):20160665. doi: 10.1098/rsif.2016.0665 (PMC5134017; doi:10.1098/rsif.2016.0665)
Supplement: Electronic Supporting Material [file rsif20160665supp1.pdf]

Electronic Supplementary Material for

Elongated Magnetite Nanoparticle Formation from a Solid Ferrous Precursor in a Magnetotactic Bacterium

Jens Baumgartner [1], Nicolas Menguy [2], Teresa Perez Gonzalez [1], Guillaume Morin [2], Marc Widdrat [1], Damien Faivre [1]

1. Department of Biomaterials, Max Planck Institute of Colloids and Interfaces, 14424 Potsdam, Germany;

2. Institut de Minéralogie et de Physique des Milieux Condensés, Unité Mixte de Recherche 7590 Centre National de la Recherche Scientifique, Université Pierre et Marie Curie, Institut de Recherches pour le Développement, Campus Jussieu, 75005 Paris, France

J. R. Soc. Interface

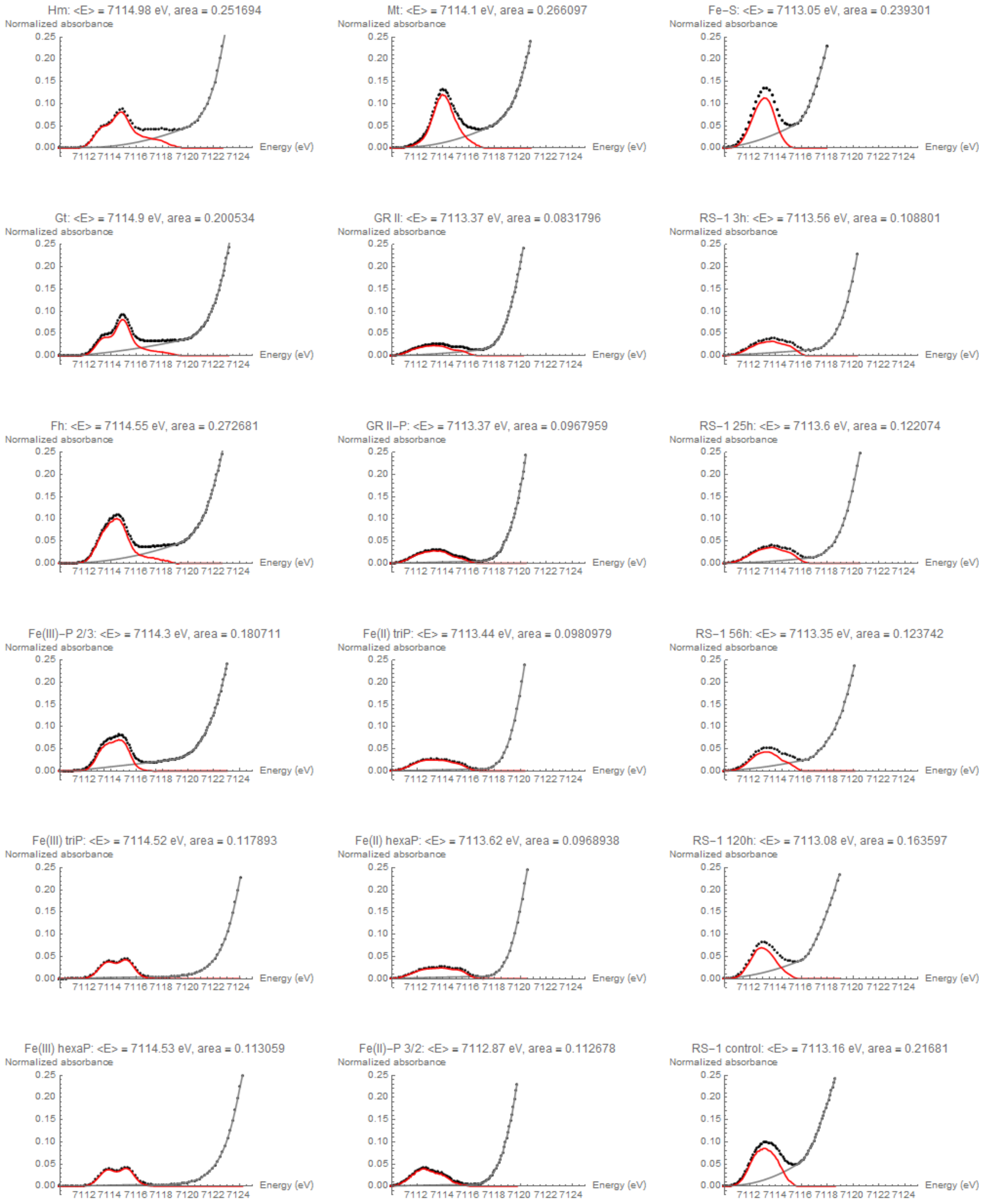

Figure S 1. Fe K-edge X-ray absorption pre-edge peaks of reference compounds and RS-1 bacteria at different time points after transfer from iron-deplete to iron-spiked medium (3, 25, 56, 120 h) or grown in conventional medium without prior iron starvation (RS-1 control). Black dots: measured data points; gray line: background (2nd order spline function), red line: background subtracted pre-edge peak used for centroid and area determination.

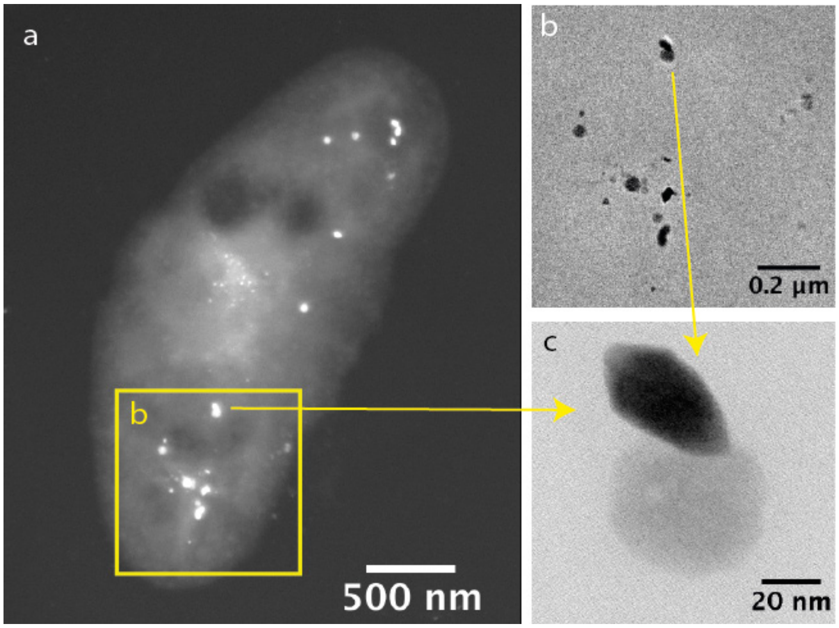

Figure S 2 . D. magneticus RS-1 cell grown continuously in the presence of iron (control) rarely also exhibits Fe-P granules. (a) HAADF-STEM of cell, (b) bright-field of marked area, (c) large magnification with bullet-shaped magnetite and spherical Fe-P granule.

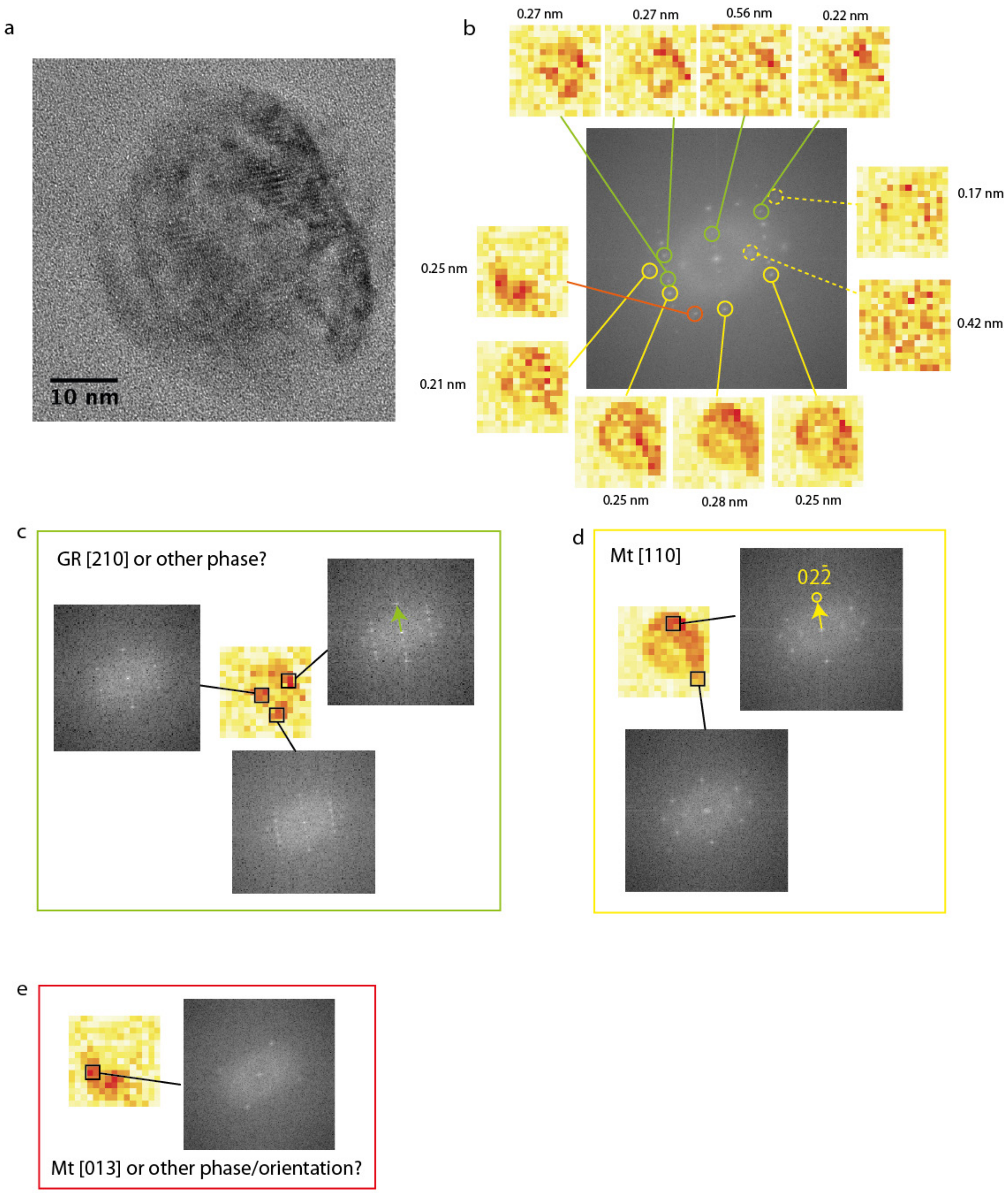

Figure S 3. (a) Polycrystalline particle with near-spherical morphology. (b) FFT of complete structure is composed of three different areas, (c) a phase with pattern similar to green rust (GR) observed [210], (d) magnetite (Mt), (e) third component with different orientation and/or phase. Arrows in (c) and (e) indicate a common direction of the unknown phase with Mt <110>. Heat maps indicate intensity of marked reflections with given spacings over the complete HRTEM image. Intensity mapping of marked reflections in FFTs (minimum yellow to maximum red). Variations in the reflection intensity within the particle indicate discrete regions with varying crystallinity and orientation.

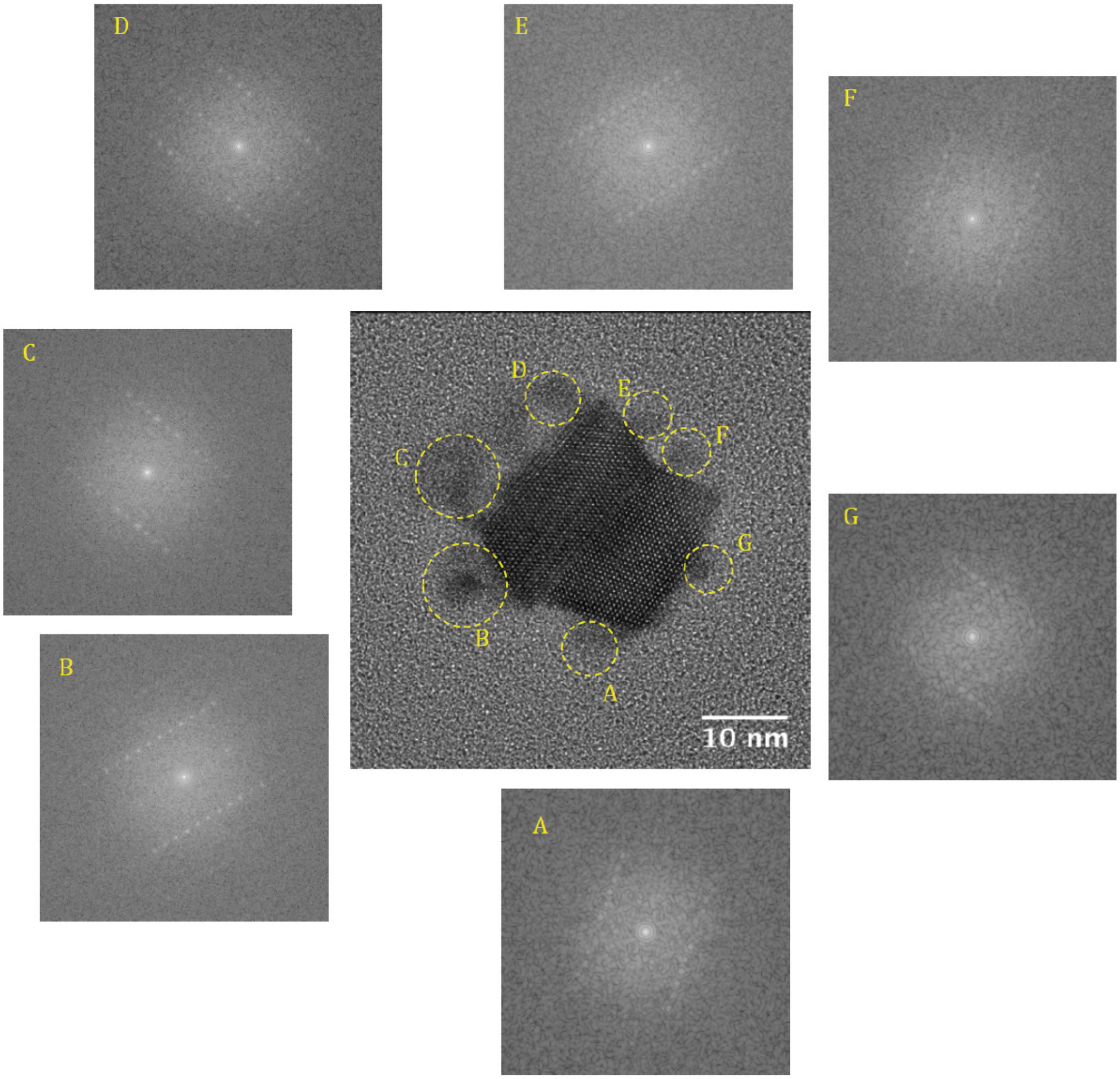

Figure S 4. Epitaxy between magnetite and surface-bound phase. Magnetite twin crystal with exposed {111} surfaces, surface-attached phase is crystallographically oriented with respect to <111> directions (FFTs).

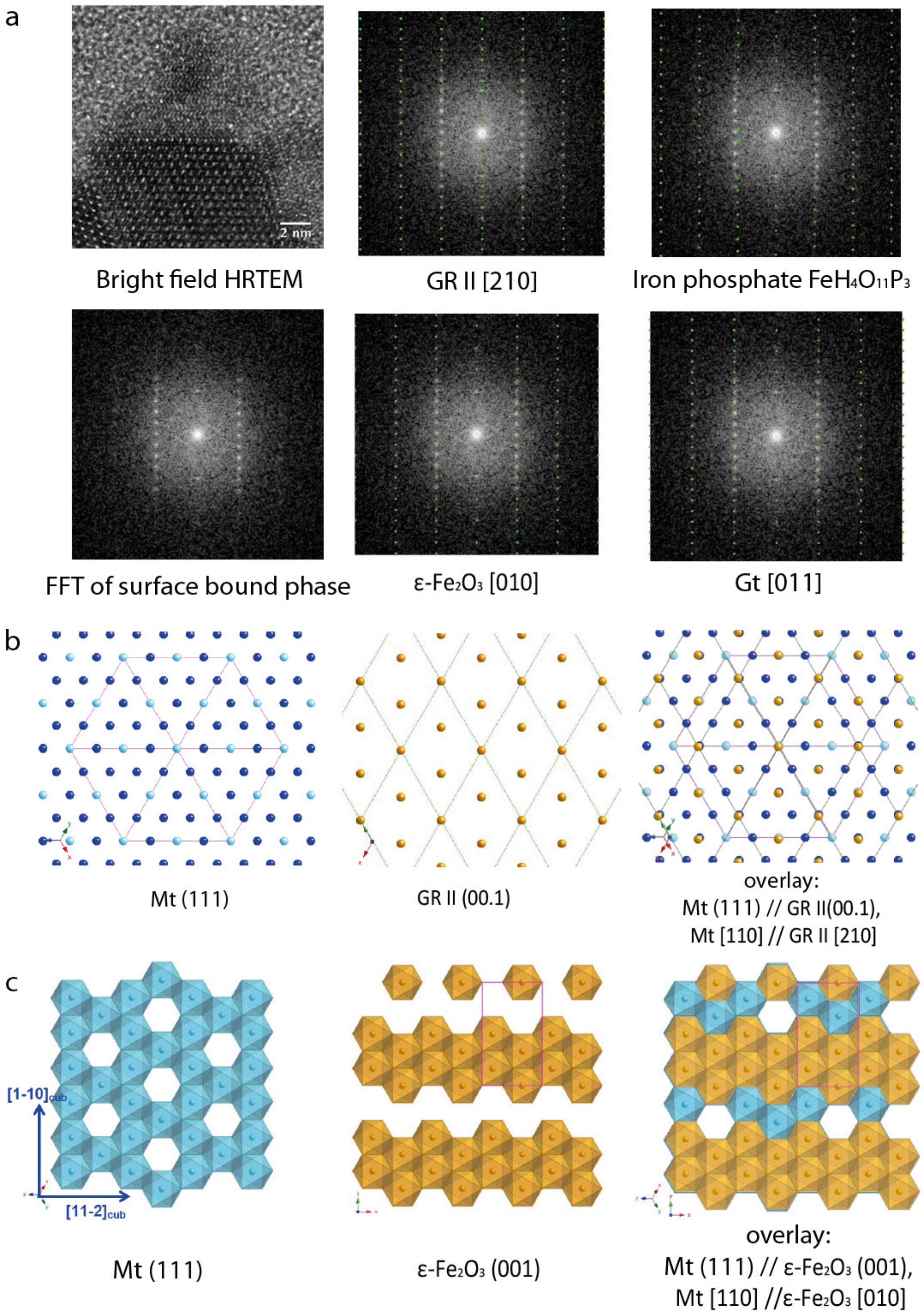

Figure S 5. Epitaxy between magnetite and surface-bound phase. (a) Bright field HRTEM micrograph of unknown attached phase, FFT and overlaid simulated small area electron diffraction patterns (green): the FFTs of surface-bound phase can be attributed to several potential phases: green rust II, ε-Fe2O3, goethite, iron phosphate and potentially others. However, epitaxy with Mt can only be achieved for GR II or ε-Fe2O3. Inferred crystallographic relationships between (b) GR II and Mt or (c) ε-Fe2O3 and Mt as determined from FFT and known crystal structure, top view of the terminating surfaces (left and middle) and the inferred interface between phases (right).

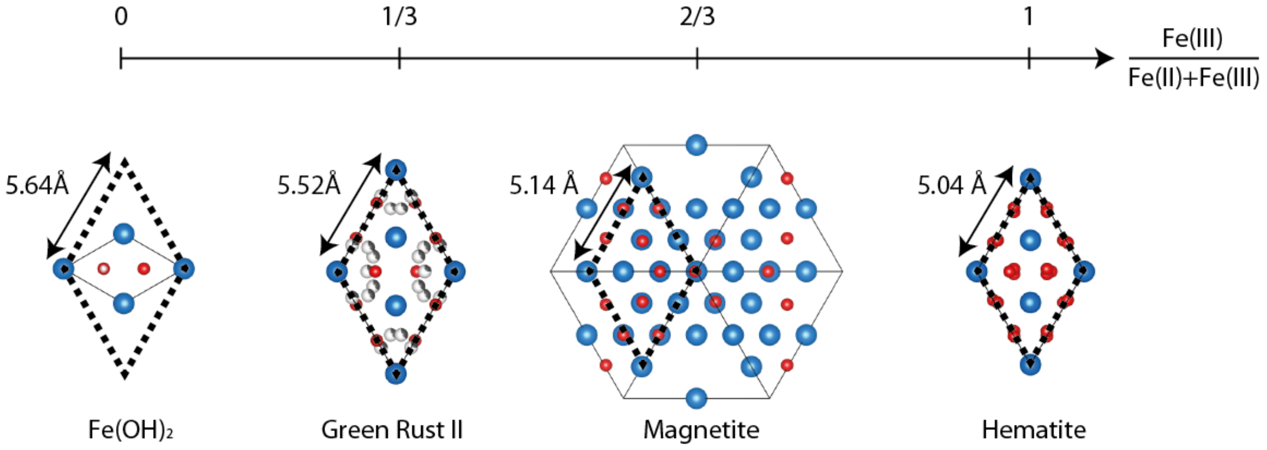

Figure S 6. Crystal structures of iron hydroxides and oxides seen along zone axes normal to the hexagonal oxygen stacking; Fe (blue), O (red), H (white). The black dotted rhombi mark the motif corresponding to the (00.1) plane in the unit cell of green rust in the other phases. The lattice contracts as a function of the relative ferric iron content. For epitaxy between two such phases, the lattice has contract correspondingly.

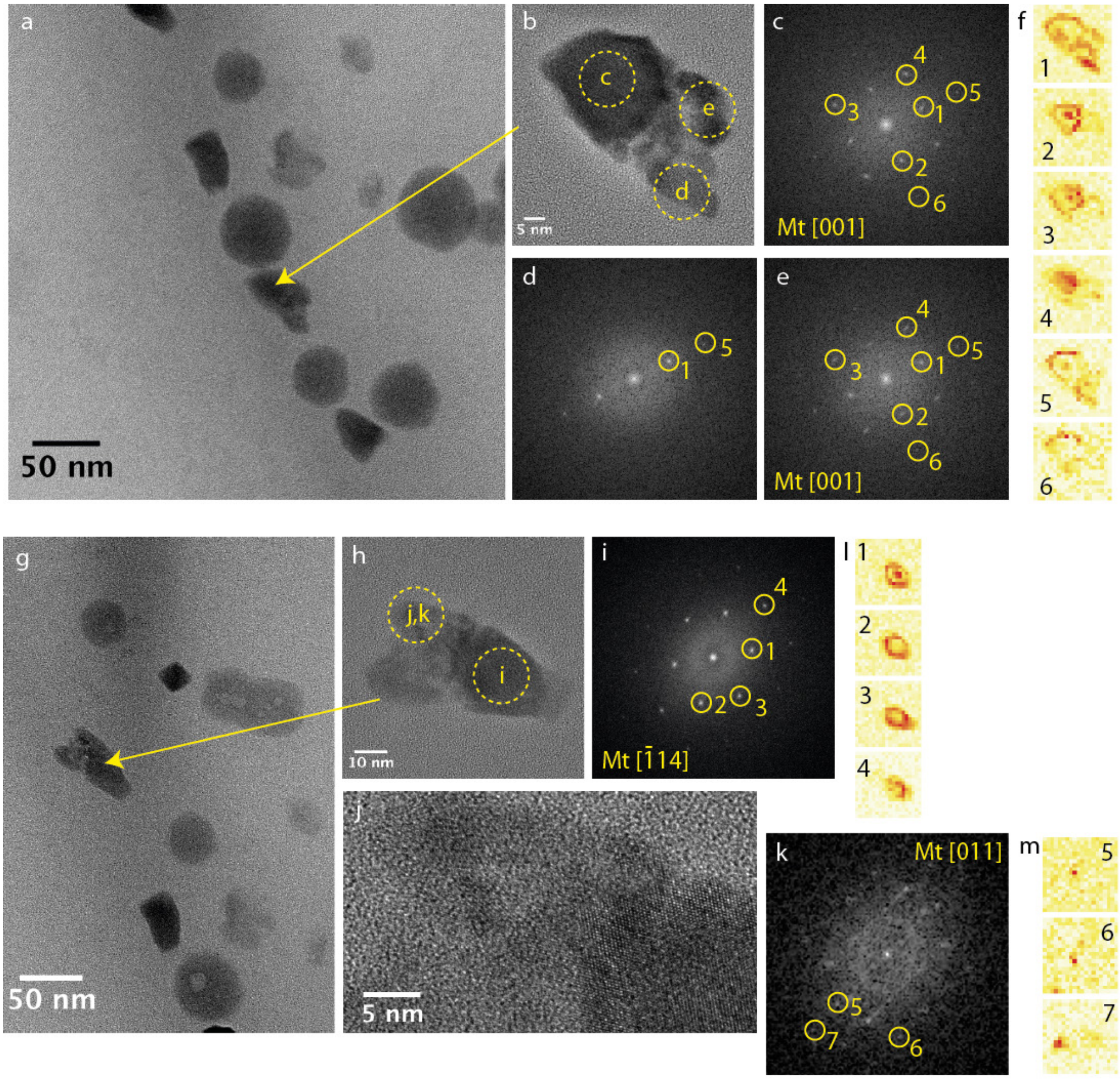

Figure S 7. Particles with morphology similar to mature magnetosomes with incomplete structure. (a, b; g,h,j) bright field TEM; here Fe-P granules appear at potentially interstitial chain sites between magnetite particles in line with a role as precursor (c-e; i,k) FFTs of marked areas, (f, l, m) intensity mapping of marked reflections in FFTs (minimum yellow to maximum red). The intensities are measured using FFTs of ~ 2.5 nm x 2.5 nm large areas mapped over whole HRTEM images (b, h). Variations in the reflection intensity within a particle indicate discrete regions with varying crystallinity and orientation.

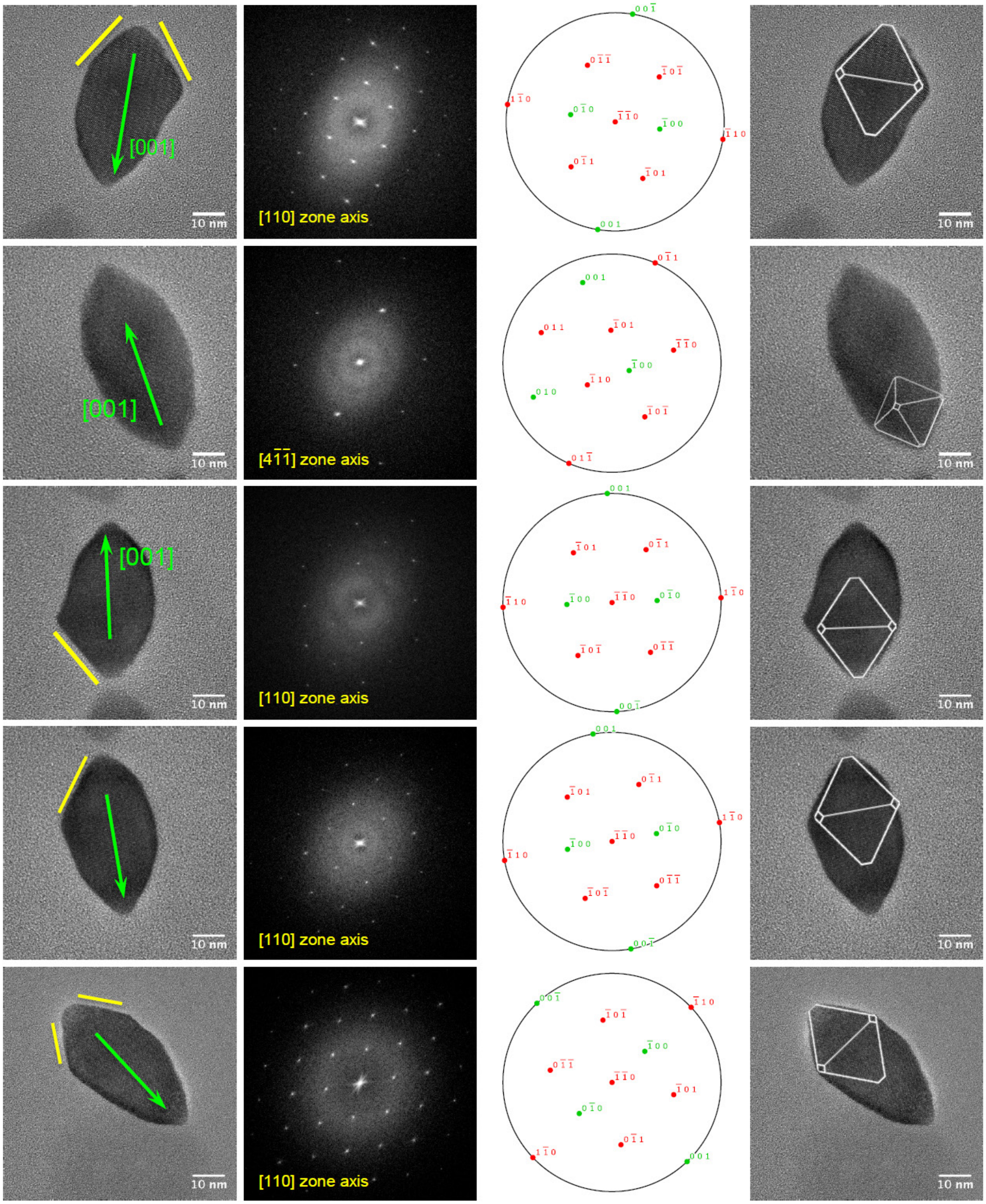

Figure S 8. Morphology of RS-1 mature magnetosomes. From left to right : Bright field HRTEM with [001] direction indicated (green arrow) and {111} planes (yellow line), FFT with indicated zone axis, corresponding pole figures and morphological relationship with the cuboctahedron.

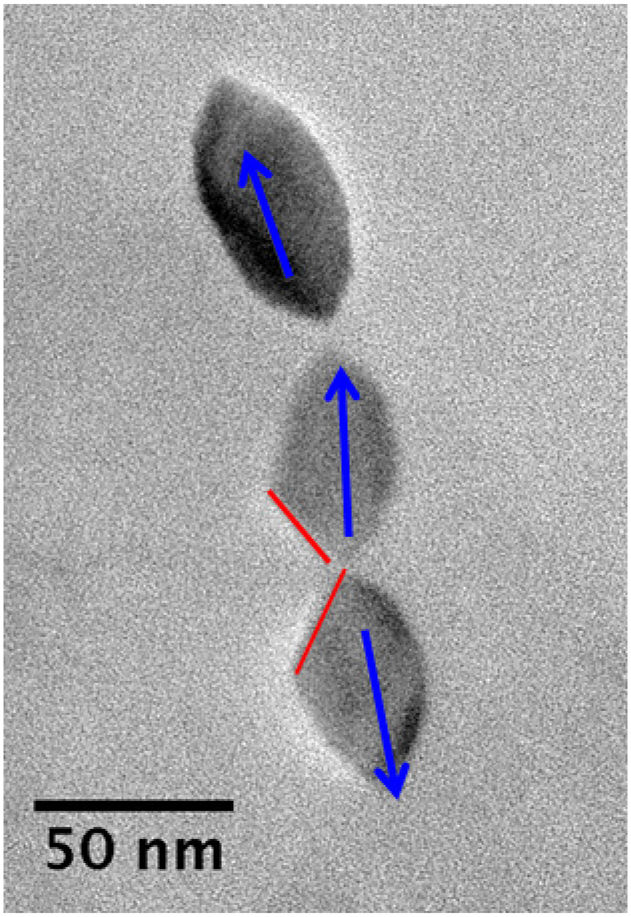

Figure S 9. Small chain of magnetosomes aligned along <001> (blue arrows), red lines indicate {111} planes.
